# Causal effect of air pollution and meteorology on the COVID-19 pandemic: A convergent cross mapping approach

**DOI:** 10.1016/j.heliyon.2024.e25134

**Published:** 2024-01-26

**Authors:** Yves Rybarczyk, Rasa Zalakeviciute, Esteban Ortiz-Prado

**Affiliations:** a School of Information and Engineering, Dalarna University, Falun, Sweden; b Universidad De Las Américas, Quito, Ecuador

**Keywords:** COVID-19, Empirical dynamic modeling, Air quality, Weather, Causation

## Abstract

Environmental factors have been suspected to influence the propagation and lethality of COVID-19 in the global population. However, most of the studies have been limited to correlation analyses and did not use specific methods to address the dynamic of the causal relationship between the virus and its external drivers.

This work focuses on inferring and understanding the causal effect of critical air pollutants and meteorological parameters on COVID-19 by using an Empirical Dynamic Modeling approach called Convergent Cross Mapping. This technique allowed us to identify the time-delayed causation and the sign of interactions. Considering its remarkable urban environment and mortality rate during the pandemic, Quito, Ecuador, was chosen as a case study.

Our results show that both urban air pollution and meteorology have a causal impact on COVID-19. Even if the strength and the sign of the causality vary over time, a general trend can be drawn. NO_2_, SO_2_, CO and PM_2.5_ have a positive causation for COVID-19 infections (ρ > 0.35 and ∂ > 9.1). Contrary to current knowledge, this study shows a rapid effect of pollution on COVID-19 cases (1 < lag days <24) and a negative impact of O_3_ on COVID-19-related deaths (ρ = 0.53 and ∂ = −0.3). Regarding the meteorology, temperature (ρ = 0.24 and ∂ = −0.4) and wind speed (ρ = 0.34 and ∂ = −3.9) tend to mitigate the epidemiological consequences of SARS-CoV-2, whereas relative humidity seems to increase the excess deaths (ρ = 0.4 and ∂ = 0.05).

A causal network is proposed to synthesize the interactions between the studied variables and to provide a simple model to support the management of coronavirus outbreaks.

## 1 Introduction

Environmental pollutants intricately interact with human health, forming a multifaceted relationship that significantly impacts overall well-being. Currently, atmospheric pollution is the greatest environmental risk factor for premature mortality and health problems in the human population [1]. Millions of lives are lost every year since 99 % of people live in areas with poor air quality [2,3]. Furthermore, less privileged populations are more exposed to air pollution (whether that be because of city- or country-scale inequality) due to a proximity to contamination sources or relaxed environmental regulations [[4], [5], [6]]. It has been proven that exposure to long- and short-term atmospheric gaseous phase (i.e., nitrogen dioxide (NO_2_), sulfur dioxide (SO_2_), carbon monoxide (CO), and ozone (O_3_)) and particulate matter ≤2.5 μm (i.e., PM_2.5_) pollutants causes an array of respiratory and cardiovascular diseases, diabetes, and psychological alterations, among other health problems [[7], [8], [9]] that, especially affect risk populations (e.g., older people, children and people with pre-existing health problems). Moreover, it has been suggested that air pollution increases the prevalence of respiratory diseases and related deaths due to the risk of increased transmission and synergetic pathogenic effects as seen during influenza and SARS outbreaks [[9], [10], [11]]. Similarly, it has been observed that meteorological parameters, such as temperature and humidity, affect the spread of respiratory viruses [12]. However, whether the effects are positive or negative varies by region: i) cold-dry weather at high latitudes, and ii) high humidity rainy seasons in warm tropical places would improve the survival rate of viral nanoparticles and increase the chances of infectivity [[13], [14], [15], [16], [17], [18], [19], [20], [21]]. Wind speed may contribute to the spread of influenza in a dual manner: i) low winds increase virus transmission from one host to another, and ii) strong winds help increase the dispersion and ventilation effects [22].

In addition to the pre-existing pressure of social and economic inequalities, climate change and environmental pollution in late 2019, the global human population encountered another zoonotic coronavirus, namely the SARS-CoV-2 virus – a microorganism responsible for COVID-19 [23]. COVID-19 took the world by surprise, affecting some regions and populations more than others [24]. These differences raised several concerns and hypotheses related to whether there might be a relationship between the COVID-19 attack rate and air quality and meteorological conditions. In response to that, a number of scientific studies have investigated how urban pollution and weather may have affected the COVID-19-related infection rates and deaths [[25], [26], [27], [28], [29], [30], [31], [32], [33]]. While the correlation between COVID-19 and the environment is influenced by various factors, including population density, health system readiness, and human mobility, emerging research suggests a direct link between the environment and an increased susceptibility to severe illness or mortality caused by COVID-19 [34]. For instance, some weather COVID-19-related research shows that temperature and humidity negatively correlate with the transmission of the virus [[35], [36], [37], [38]]. However, a study investigating a wide range of countries in both hemispheres found that the impact of temperature and humidity is non-linear and may vary depending on the country [39]. The effect of wind speed is more consistent, with most of the studies pointing toward a negative correlation with the spread of COVID-19 [31,37,40]. Air quality and COVID-19-related studies have concluded that atmospheric conditions such as air pollution may affect COVID-19 cases, making poor air quality an additional co-determinant of COVID-19-related mortality [41]. Both short-term and long-term exposure to air pollution have been associated with elevated infection rates and increased mortality related to COVID-19 [26]. In terms of specific pollutants, extensive research has revealed that PM_2.5_ (particulate matter with a diameter of 2.5 μm or less) and NO_2_ (nitrogen dioxide) play a significant role in influencing the incidence of COVID-19 infections and mortality [[41], [42], [43], [44], [45], [46], [47]]. These pollutants are believed to exert their impact through synergy, leading to altered inflammatory states, elevated oxidative levels, and overreactive immunological responses. For example, a recently published study suggests that an increase in PM_2.5_ by just 1 μg m^−3^ might increase the number of COVID-19 deaths by 8 % [48]. A 10-μg/m^3^ increase (lag 0–14) in PM_2.5_, PM_10_, NO_2_, and O_3_ was also associated with a 2.24, 1.76, 6.94, and 4.76 % increase in the daily counts of COVID-19 cases, respectively [49].

While such studies provide valuable insights, it is important to note that most of them utilized simple correlation methods and cross-sectional data analyses. As a result, they do not establish a causal relationship between pollution and COVID-19 cases or mortality. There are very few studies that investigated non-linear relationships between the air pollutants and the COVID-19 infection or mortality rates. The Generalized Additive Model (GAM) approach was applied to determine the lagged effect of six criteria air pollutants on the daily COVID-19 confirmed cases [49]. In a separate study, an investigation was conducted to explore the relationship between PM_2.5_ and COVID-19 mortality, employing a negative binomial mixed model [50]. However, these models can only identify associations between variables; they do not establish a causal link between them. To the best of our knowledge, only one study has employed a Causal Direction from Dependency (D2C) algorithm to check the relationship between air pollution and COVID-19; and it was conducted in three French cities [41]. The core of their approach is the use of Artificial Neural Networks (ANNs) to link PM_2.5_, CO_2_, NO_2_, and COVID-19 deaths; however, machine learning algorithms are adapted for prediction and are not specifically for the inference of causation.

In this manuscript, we present the first comprehensive work on the effect of five criteria pollutants (i.e., NO_2_, SO_2_, CO, O_3_ and PM_2.5_) and three selected meteorological parameters (i.e., temperature, wind speed and relative humidity) on the COVID-19 pandemic through a data-driven modeling approach called Convergent Cross Mapping (CCM). This empirical method is specially designed to explore the dynamic of causal relationships within a complex ecosystem, where factors interact in a non-linear manner [51]. CCM is a powerful methodological approach that can help distinguish causality from spurious correlation in time series from dynamic systems. The technique is based on the idea that causation can be established if states of the causal variable can be recovered from the time series of the affected variable [52]. It has several advantages over other methods for inferring causality, such as: (i) handling non-linear and non-stationary systems, where traditional approaches based on correlation or Granger causality may fail; (ii) detecting causal influences that are synergistic or masked by noise, where the variables may appear uncorrelated; (iii) distinguishing between direct and indirect causation; and, last but not least, (iv) inferring causality from observational data without the need for controlled experiments or interventions. However, it is also important to be aware of the main limitations of CCM, which are: (i) collecting enough data to reconstruct the state space of the system; and (ii) assuming that the analyzed system is deterministic and non-linear.

Keeping in mind the strengths and conditions of applying CCM, this method appears to be well-suited for examining the interplay between environmental factors (weather and pollution) and epidemiological variables (new COVID-19 cases and COVID-19-related deaths) analyzed in this study. The city of Quito in Ecuador was selected as a noteworthy case study due to its remarkable characteristics, including an exceptional elevation (the highest capital in the world), which contributes to a degradation of urban air quality [53]. Additionally, Quito's inclusion as a case study is influenced by the significant impact of COVID-19 in terms of excess deaths and overall mortality in the country [54].

## 2 Material and method

### 2.1 Study site and data recollection

Ecuador, with an area of more than 283,000 km^2^, is the smallest country in the Andean mountainous region of South America. The country is divided into four geographical regions: the coast, the highlands, the Amazon region, and the Galapagos Islands. The political division includes 24 provinces: ten in the highlands, seven on the coast, six in the Amazon region, and one in the insular region of Galapagos. By the end of 2020, the country of 17 million people had reported over 200,000 cases and 23,8000 deaths from COVID-19 [55]. Thus, in this study we will focus on the period during the first 15 months of the pandemic: March 13, 2020–June 30, 2021, where different stages of quarantine restrictions were included, but where the effect of the vaccines had not yet kicked in.

At an elevation of 2850 *m* above sea level (m.a.s.l.), on the flanks of Pichincha Volcano (elev. 4800 *m*. a.s.l.), the capital Quito spreads between the western and eastern branches of the Andean Cordillera. The Metropolitan District of Quito (DMQ), situated right on the equatorial line, extends over 40 km through a variety of elevations from over 3000 *m*. a.s.l. Toward the lower inner Andean valley (approx. elev. 2300 *m*. a.s.l.). The DMQ has almost 3,000,000 inhabitants [56] and is experiencing a rapid growth in the amount of motorized traffic that runs on poor-quality fuels (i.e., EURO 2–3), which affects air quality considerably [6,53].

### 2.2 Data source

The concentrations of criteria pollutants (NO_2_, SO_2_, CO, O_3_, PM_2.5_) and meteorological parameters (i.e., temperature – Temp, wind speed – WS, and relative humidity – RH) were retrieved from the environmental monitoring network of the Secretariat of the Environment of DMQ. Out of nine existing study sites, distributed across the long and narrow city, data were analyzed from the central Belisario (elev. 2835 *m*. a.s.l, coord. 78°29′48″W, 0°11′4.57″S) site, as it is the most representative urban site. The data of atmospheric parameters were collected through the methods in accordance with the guidelines of the United States Environmental Protection Agency [57], previously described in detail elsewhere [6]. The ground-based monitoring data were collected during the period of March 13, 2020 to June 30, 2021 so as to include the various stages of quarantine regulations, as well as after they were removed.

The official data on the number of COVID-19 cases were generated by the Ministry of Health (MoH) through the epidemiological surveillance system (ViEPI). The MoH shared these data with our research team, which enabled us to conduct a daily analysis of both COVID-19 cases and deaths. COVID-19-related excessive mortality was calculated with daily, weekly, and monthly resolution in all provinces of Ecuador. However, in this study only the daily data for the capital city were used, resulting in a dataset with a total of 475 observations.

### 2.3 State Space Reconstruction

An empirical dynamic modeling method was used to study the causal effect of air pollution and meteorology on the COVID-19 pandemic in order to capture the dynamic relationship between the environmental variables and the disease. The method is based on State Space Reconstruction (SSR) [58,59]. SSR is preferred to the Granger causality approach [60,61], because it is more appropriate for the analysis of deterministic systems [62] and complex ecological interactions [51].

**Fig. 1:**
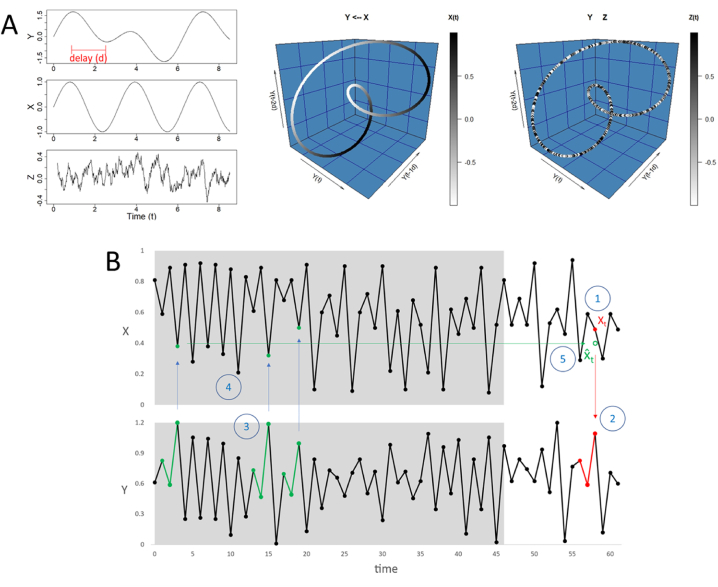
(**A**) State Space Reconstruction (SSR) method to test the causal relationship between two variables. This example looks for a possible effect of X and Z on Y. A time series displays the original dynamic of each variable. The continuous mapping (gradual transitions in shade) between X and the tridimensional Y delay vector (red segment) indicates that X causes Y. Conversely, the cross mapping between the SSR of Y and the corresponding values of Z results in a bumpy shading, suggesting that Z has no causal effect on Y. (**B**) The CCM principle for testing if X causes Y consists of predicting X from Y and assessing the accuracy of the prediction (xmap skill). The main steps of the cross mapping are: (1–2) matching the Y delay vector (red segments) with the X value (X_t_) to predict (red dot); (3) identifying the most similar Y delay vectors (green segments) in the training set (grey area); (4) looking up to the corresponding points in the time series X (green dots); and (5) computing their weighted mean to get the predicted value X^_t_ (green empty dot). (For interpretation of the references to colour in this figure legend, the reader is referred to the Web version of this article.)

The SSR technique can be explained through a dynamic ecosystem with three variables – X, Y, and Z – which can potentially have a causal relationship to each other. To detect the causation, a multidimensional state space of Y is built from recent and past values of this variable (called ‘delay space’). Fig. 1A represents a tridimensional reconstruction of the Y time series using its current (Y_t_) and two delay values (Y_t-1d_ and Y_t-2d_). Each measurement (3D-coordinates of a point or ‘delay vector’) of Y is then shaded from the concurrent value of the other variable to be tested (X or Z). If a homogenous gradient is obtained, it means that concomitant points in the delay space of Y correspond to similar values of X (or Z). Such a ‘continuous delay map’ allows us to infer that Y is causally driven by X. Contrary to this, a bumpy shading suggests an absence of causal relationship between two variables.

SSR is based on Takens' theorem [63], which predicts a continuous delay map from driven to driving factors in a coupled deterministic dynamic system. In that sense, the notion of ‘dynamic driving’ is assimilated as causation. The SSR method consists of detecting a continuous delay map between two variables and inferring causation, considering that a continuous delay map from Y to X indicates that X causes Y, and not the contrary [51].

In practice, SSR is very sensitive to the quality of data measurement. For instance, identifying continuity can be challenged by the presence of noise [62]. However, several techniques have been developed to overcome this limitation [59]. The most popular of them is Convergent Cross Mapping (CCM). This method assesses the capacity of a variable to predict the actual value of another variable through a metric called ‘cross map skill’ (xmap). The concept is similar to the homogenous gradient shading described in Fig. 1A.

The example presented in Fig. 1B illustrates how CCM is computed to test if X causes Y. The principle consists of predicting X from Y by splitting the data into training and testing sets. First, consider a point (X_t_) in the testing set of the time series X. Second, search for the simultaneous delay vectors (Y_t_, Y_t-1d_, Y_t-2d_) in the time series Y. Third, detect the most similar pattern of Y delay vectors in the training set. Fourth, look up to the concurrent points in the X series. Fifth, calculate the weighted average of the selected points in the training set to get a predictive value of X_t_. The process is then repeated for the other values in the testing set. Finally, the cross map skill is obtained by computing the Pearson coefficient of correlation (or similar metrics) between predicted (X_t_) and actual (X_t_) values of X. The best delay (d) and vector length (or embedded dimensions – E) are defined through a grid search, varying ‘d’ and ‘E’ from 1 to 10, in order to get the combination that maximizes the prediction skill.

**Fig. 2:**
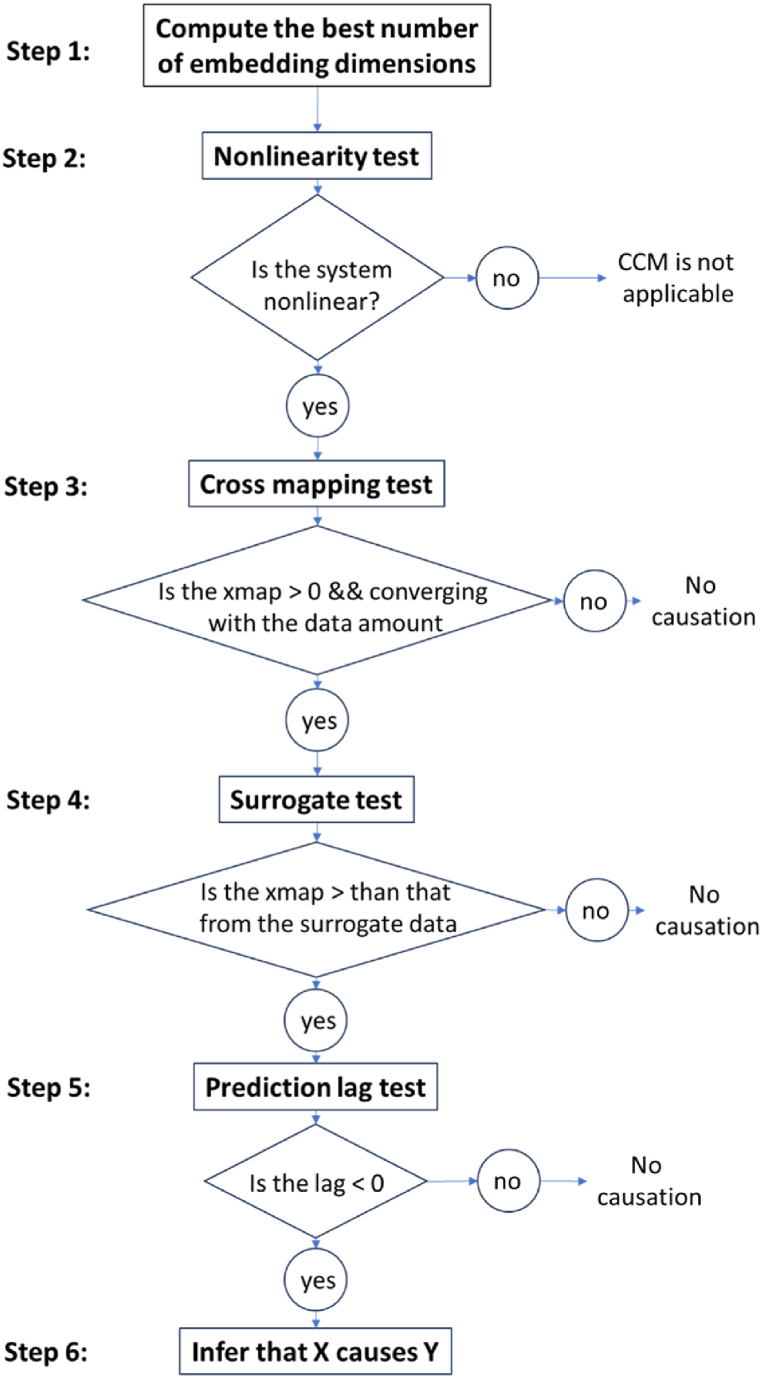
Algorithm defining the different steps applied to infer a CCM-based causation between the studied variables.

Four criteria have been established to reduce the risks of failure (false positive and false negative) in inferring causation [62,64]. First, the cross map skill should be positive. Second, increasing the library size (i.e., amount of training data) should improve the prediction. Third, the xmap should be statistically significant according to a surrogate data test. In this study, the surrogate is created from the original data by randomizing the phases of a Fourier transform [65]. Fourth, the xmap should be maximized at a negative lag, because the effect of a variable on another occurs with a certain delay. The lag days of the causations are particularly relevant in this work and will be analyzed, because the effect of the independent variables (weather and atmospheric pollution) on the dependent variables (COVID-19 cases and excess deaths) is not supposed to be instantaneous. A graphical representation of the different steps that allowed us to infer a causation according to the CCM approach is depicted in Fig. 2.

There are several steps in the present study (see Appendix A - Fig. A1 for a visualization of the workflow). A preliminary task focuses on identifying and selecting the most relevant variables in order to proceed with a pairwise causation analysis. Based on the results, it will be possible to distinguish between causal and non-causal dependencies and even, in some cases, eliminate irrelevant variables. When a causation is detected, a further analysis will involve determining the time delay of the causal relationship, which is the lag day corresponding to the highest cross map skill. According to the peak size and its respective delay of occurrence, it will be deduced whether the causation is direct or indirect [64]. A direct causality is identified by a higher and more recent xmap peak than an indirect causal dependency. Also, the sign of the causation between each variable (positive vs negative) is obtained by computing the *S*-map coefficient, which measures the time-varying intervariable interaction strength estimated by the *S*-mapping method as partial derivative [66]. The final objective is to build a causal network model of the most plausible interactions and respective dynamic between all the variables considered in the study.

The analyses are carried out using the rEDM library, a free software package written in the R language [67]. The function EmbedDimension (.) gave us the optimal number of dimensions to predict COVID-19 cases and excess deaths as E = 10 and E = 7, respectively. Each pairwise causation was analyzed over a period of 71 lag days: 10 positive lags (future), 1 instantaneous (present), and 60 negative lags (past).

## 3 Results

### 3.1 Interaction between air pollution and COVID-19

**Fig. 3:**
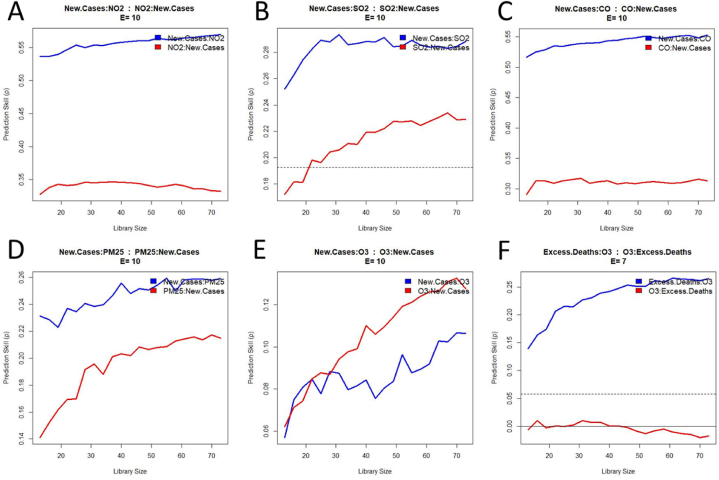
Convergent cross mapping of air pollution with new cases of COVID-19 and excess deaths. When new cases ‘xmap’ an air pollutant (New.Cases:Pollutant), it indicates the skill for the pollutant to cause COVID-19 (blue curve). Conversely, when an air pollutant ‘xmap’ new cases (Pollutant:New.Cases), it indicates the skill for COVID-19 to cause the pollutant (red curve). The same principle is applied to the extra deaths (panel F). The dotted line (panel B and F) indicates the coefficient of correlation between the two variables (not visible on panel A, C, D, and E because of a too low value). (For interpretation of the references to colour in this figure legend, the reader is referred to the Web version of this article.)

The CCM analysis shows that the cross map skill between air pollutants and new cases of COVID-19 is positive (Fig. 3A-E). This result meets the first criterion to infer a causation between these variables. The cross mapping ‘New.Cases:Pollutant’ (Fig. 3A-E, blue curves) is higher than the cross mapping ‘Pollutant:New.Cases’ (Fig. 3A-E, red curves) for all the pollutants with the exception of O_3_, which suggests that pollution causes the COVID-19 cases and not the opposite. In addition, the prediction skill of the xmapping ‘New.Cases:Pollutant’ increases with an increasing amount of training data (Library Size), which is not always the case for the inverse xmapping (Fig. 3. A and 3. C). This outcome meets the second criterion of causation.

**Fig. 4:**
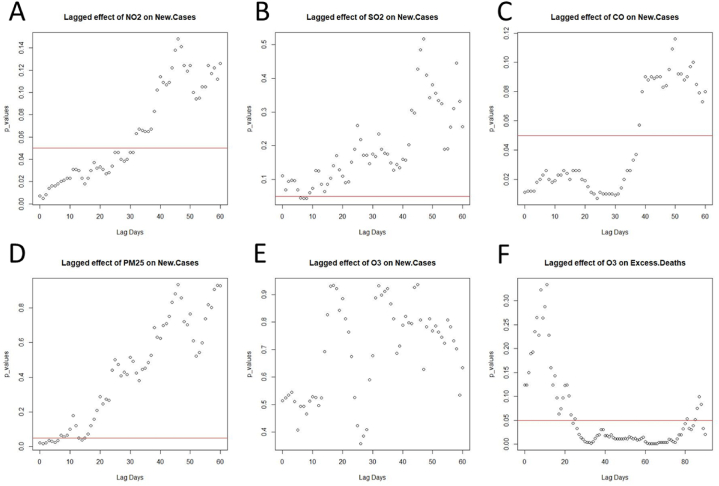
Statistical analysis of the cross map skill difference between the original time series and a surrogate dataset for several lag days. Values below p = .05 (red line) indicate a significant difference. (For interpretation of the references to colour in this figure legend, the reader is referred to the Web version of this article.)

The surrogate data tests of the xmap between each pollutant and new COVID-19 cases were performed over 60 days (Fig. 4A-E). The results show a significant difference between the surrogate and the original time series for all the studied air contaminants with the exception of O_3_. This difference remains significant over the first month for NO_2_ and CO and during the first two weeks for PM_2.5_ and SO_2_ (Fig. 4A-D). The fact that the third criterion is met for NO_2_, CO, PM_2.5_, and SO_2_ confirms the potential effect of these pollutants on the increase of the infection. Since O_3_ failed this test, it cannot be considered a causal factor of COVID-19 cases. Such a deduction is supported by the fact that the xmap skill of ‘New.Cases:O3’ is lower than O3:New.Cases (Fig. 3E).

However, the CCM analysis between O_3_ and excess deaths provides a different outcome. As shown in Fig. 3F, the prediction skill of ‘Excess.Deaths:O3’ (i) is positive, (ii) increases with the library size, and (iii) is higher than ‘O3:Excess.Deaths’ (ρ ≈ 0). This effect is confirmed by the surrogate test, which shows a significant difference from 20 to 80 days of lag time (Fig. 4F). Altogether, these results suggest a causal impact of O_3_ on the mortality rate during the pandemic.

So far, the CCM analysis shows that the five criteria pollutants have a potential causal effect on COVID-19, with four primary pollutants involved in the number of new cases (NO_2_, CO, PM_2.5_, and SO_2_) and one secondary pollutant impacting the excess deaths (O_3_). The confirmation of these causal relationships depends on the results of the prediction lag test. To meet this last criterion, the cross map skill should be higher for predicting past than future values. Table 1 (see also Appendix B - Fig. B1) shows that all the cross mappings have a peak value of prediction skill occurring in the past. The time delay of the highest xmap skill for ‘NO2 - > COVID-19 cases’, ‘PM2.5 - > COVID-19 cases’ and ‘SO2 - > COVID-19 cases’ is shorter (less than 10-day lag) than the one for ‘CO - > COVID-19 cases’ and ‘O3 - > Excess deaths’ (more than 20 days). It is also worth noticing that the value of these peaks is higher for ‘NO2 - > COVID-19 cases’, ‘CO - > COVID-19 cases’ and ‘O3 - > Excess deaths’ (ρ > 0.5) than ‘PM2.5 - > COVID-19 cases’ and ‘SO2 - > COVID-19 cases’ (ρ < 0.4). These two latter pollutants are known to be more difficult to predict than the other three [68]. This last outcome validates the assumption of a causal impact of NO_2_, CO, PM_2.5_, and SO_2_ on the new cases of COVID-19 and O_3_ on the excess deaths during the pandemic.

**Table 1:** The value and corresponding time delay of the highest cross map skill for each causal interaction, classified in ascending order of lag days.

Causation	Highest Xmap Skill (ρ)	Lag Day of the ρ Peak
NO2 - > COVID-19 cases	0.63	1
PM2.5 - > COVID-19 cases	0.36	1
Temp - > COVID-19 cases	0.23	2
SO2 - > COVID-19 cases	0.36	7
CO - > COVID-19 cases	0.58	24
O3 - > Excess deaths	0.53	33
Temp - > Excess deaths	0.24	58
RH - > Excess deaths	0.40	>60
WS - > Excess deaths	0.34	>60

### 3.2 Interaction between meteorology and COVID-19

**Fig. 5:**
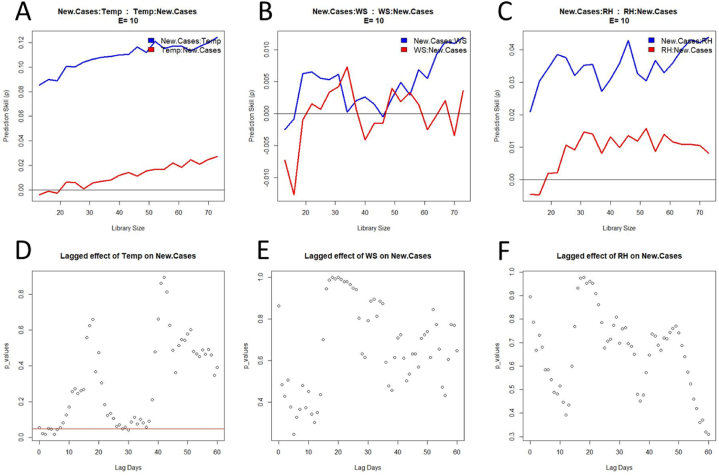
Convergent cross mapping (top panels) and respective surrogate data test (bottom panels) for inferring the causal dependency between meteorological factors and COVID-19 cases. The red line on panel D indicates the threshold (p = .05) for a significant difference. (For interpretation of the references to colour in this figure legend, the reader is referred to the Web version of this article.)

The cross mapping ‘New.Cases:Temp’ (Temperature) is positive and increases with the library size, whereas the opposite xmap (Temp:New.Cases) is almost null (Fig. 5A). The surrogate test for ‘New.Cases:Temp’ shows a significant difference mostly over the first week (Fig. 5D). These two results suggest a causal effect of temperature on COVID-19 cases, which has to be confirmed by the prediction lag test. On the other hand, the CCM analysis of the relationship between the two other meteorological factors (Wind Speed – WS – and Relative Humidity – RH) and COVID-19 cases indicates a weak causation (Fig. 5. B and 5. C). Even if the xmap ‘New.Cases:WS’ and ‘New.Cases:RH’ is positive, the prediction skill is close to zero and can hardly converge to a significantly high value (noisy evolution of the xmap skill with an increasing amount of training data). This observation is confirmed by the surrogate test, which reveals no significant difference whatever time delay is considered (Fig. 5E and 5F). It can be inferred from this result that WS and RH do not cause new COVID-19 cases.

**Fig. 6:**
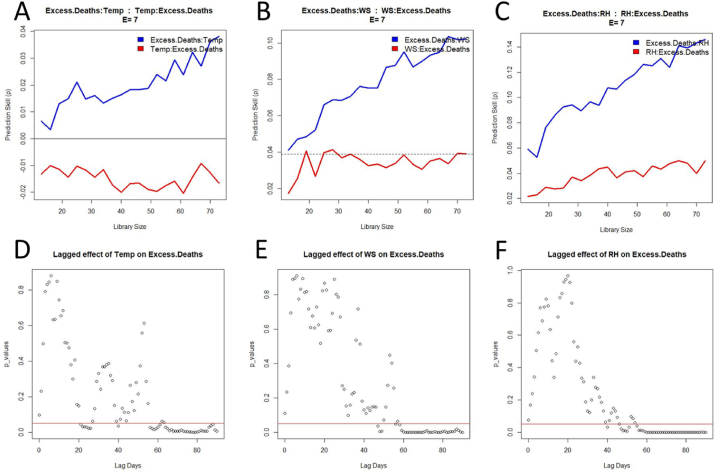
Convergent cross mapping (top panels) and respective surrogate data test (bottom panels) for inferring the causal dependency between meteorological factors and excess of deaths. The dotted line (panel B) indicates the Pearson coefficient of correlation between the two variables (not visible on panel A and C because of a too low value). The red lines on the bottom panels show the threshold (p = .05) for a significant difference. (For interpretation of the references to colour in this figure legend, the reader is referred to the Web version of this article.)

Considering that two out of three meteorological factors did not have a causation on the COVID-19 cases, an additional analysis consisted of researching a possible effect on the excess deaths. For this interaction, the three meteorological variables exhibit a similar profile for both the cross mapping test and the surrogate test (Fig. 6). The xmapping ‘Excess.Deaths:Temp’, ‘Excess.Deaths:WS’, and ‘Excess.Deaths:RH’ provide (i) a positive xmap skill and (ii) a prediction skill increasing with the library size. The opposite xmapping give a poor prediction, considering that the values are negative (Fig. 6A), below the Pearson coefficient (Fig. 6B), or almost null (Fig. 6C). Such a result suggests a unidirectional causation from meteorological factors to mortality rate. This hypothesis is supported by the surrogate data test showing a significant difference from about 50 days of lag time (Fig. 6D-F).

The prediction lag test confirms the fact that temperature causes both COVID-19 cases and the excess deaths, and the causal effect of the three meteorological factors on the mortality rate during the pandemic (Table 1 and Appendix B - Fig. B2). The impact of temperature on the new cases was rapid, with a lag time of only two days. Meanwhile, the causal dependency with the extra deaths was slower, taking about two months to reach the highest prediction skill. Considering the delayed death of an infected individual, the expectation is that a causal relationship will be observed with the excess deaths occurring later.

### 3.3 Time-delayed causality

The time-delayed causation is determined by the number of lag days corresponding to the highest value of xmap skill. The previous analyses identified a total of nine causal relationships, divided into five and four causal effects on COVID-19 cases and excess deaths, respectively. The first observation shows that the latency of the causation is shorter for COVID-19 cases than mortality regardless of the impacting factor (Table 1). The delay window ranges from 1 to 24 lag days. Most of the variables (four out of five) have the maximum effect on COVID-19 cases over the first week. CO is the pollutant with the longest delay, because this gas tends to persist in the atmosphere for a long period of time. Another particularity is the fact that the prediction skill is higher for the air contaminants (0.35 < ρ < 0.64) than for the meteorological parameter (ρ = 0.23 for ‘Temp - > COVID-19 cases’).

Since people get sick before dying, the expectation was that there would be a longer delay for extra deaths than cases. As with COVID-19 infections, air quality seems to have a stronger and quicker effect than meteorology on the mortality rate. After one month, the prediction skill for ozone is ρ = 0.53, whereas the xmap skills do not exceed 40 % (RH) for the meteorological factors with 60 days of lag time. These results (long delays and low peaks) suggest an indirect causation of the weather conditions on COVID-19-related deaths. In contrast, air pollution seems to have a direct impact on both infection and mortality.

### 3.4 Signs of interaction

**Fig. 7:**
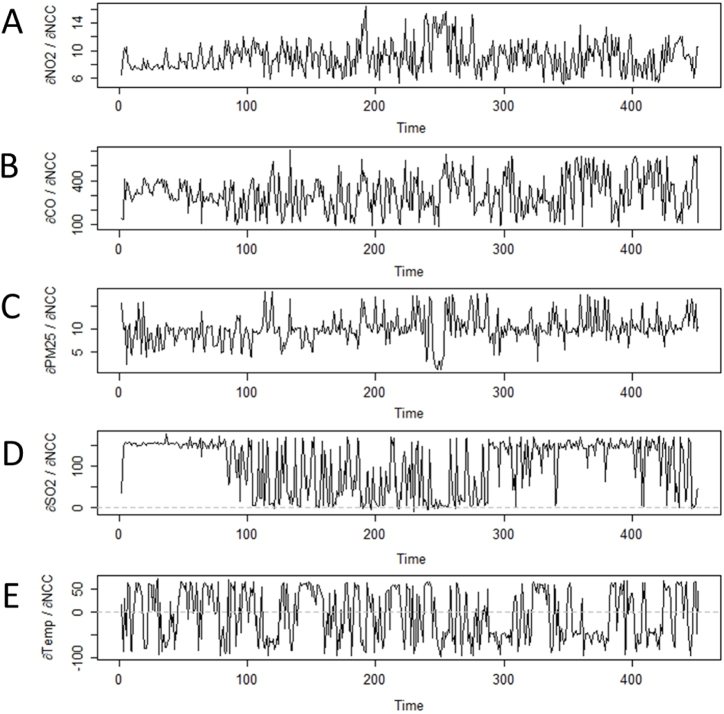
Time-varying intervariable interaction strengths for each cause of New COVID-19 Cases (NCC), computed from the *S*-map method as partial derivatives. Panels A, B, C, D and E represent the variation of the interaction for NO_2_, CO, PM_2.5_, SO_2_ and Temperature, respectively. The broken line distinguishes positive from negative *S*-map coefficients.

The *S*-mapping analysis shows a fluctuation in the interaction strength during the studied period. This variation is the signature of a dynamic system, which can exhibit an inversion of the sign of the *S*-map coefficient over time. Nevertheless, several consistencies can be extracted regarding the variable interaction with each epidemiological parameter. In terms of the new COVID-19 cases, it can be noted that the *S*-map coefficient is always positive whatever pollutants are being considered (Fig. 7). The mean coefficients for the air contaminants are as follows: NO_2_ = 9.1 (SD = 2.1), CO = 313.7 (SD = 121.7), PM_2.5_ = 10.1 (SD = 3.2), SO_2_ = 102.4 (SD = 63.8). These results indicate that the greater the level of pollution, the greater the number of COVID-19-related cases is. On the other hand, the sign of the *S*-map coefficient for temperature changes over time, with several switches from positive to negative values. Even so, the average coefficient for this meteorological parameter is negative (μ = −7.4; SD = 55.3), which suggests that the number of COVID-19 cases tends to decrease when the temperature rises.

**Fig. 8:**
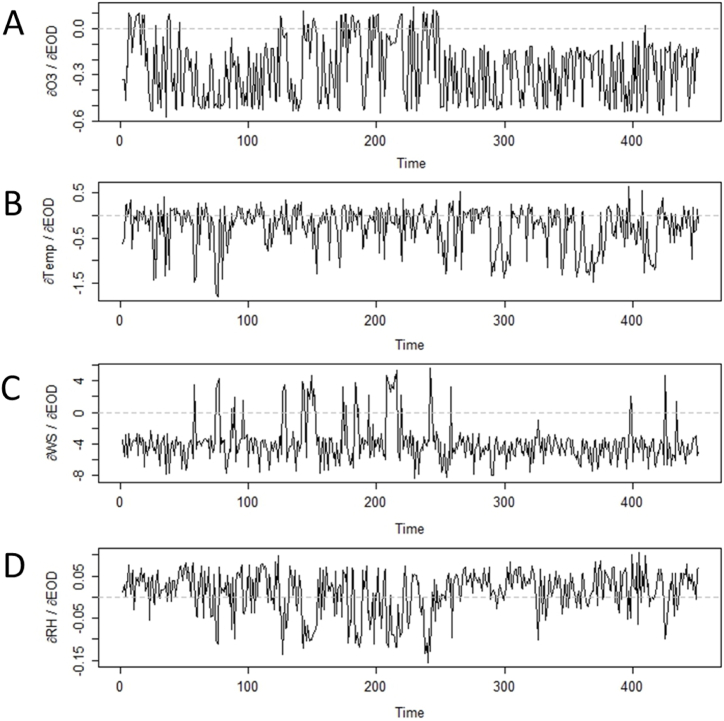
Time-varying intervariable interaction strengths for each cause of Excess Deaths (EOD), computed from the *S*-map method as partial derivatives. Panels A, B, C and D represent the variation of the interaction for O_3_, Temperature, Wind Speed and Relative Humidity, respectively. The broken line distinguishes positive from negative *S*-map coefficients.

The reading of the interaction strength with the excess deaths is less straightforward than in the case of the COVID-19 cases. All presented variables exhibit positive and negative values of *S*-map coefficients during the pandemic (Fig. 8). Nonetheless, most of these variables (three out of four) have a negative effect on the excess deaths. The computation of the partial derivatives provides the average coefficients as follows: O3 = −0.3 (SD = 0.1), Temp = −0.4 (SD = 0.4), WS = −3.9 (SD = 2.5). This outcome suggests that the higher the concentration of ozone, temperature and wind speed, the lower the number of COVID-19-related deaths. The *S*-map coefficient of RH is more difficult to interpret since the evolution of the time series aligns on the x-axis. However, the mean value for this meteorological variable is positive (μ = 0.01; SD = 0.05), which suggests that the excess deaths tends to increase with a higher percentage of RH.

## 4 Discussion

This work addresses the causal effect of air pollution and meteorology on the COVID-19 pandemic in Quito, the capital city of Ecuador. Since the case study is a complex ecosystem that is constantly evolving as a result of non-linear interaction of several variables, an empirical dynamic modeling approach was applied. The causal relationship between the independent (pollution and meteorology) and dependent (epidemiological parameters) variables was inferred from a Convergent Cross Mapping (CCM). This method involved reconstructing the state space (SSR) of an epidemiological variable (Epi) from the lagged version of its time series and comparing this reconstruction to the SSR of an environmental variable (Env). The assumption is that if ‘Env’ causes ‘Epi’, then states of ‘Env’ must be recovered from the history of ‘Epi’ [51]. This technique allowed us to distinguish causality from simple correlation and to detect causal relationships between an environmental parameter and a COVID-19 marker sharing the same dynamic system, even if they may be weakly correlated.

The result of our analysis shows that air pollution has a causal effect on COVID-19 infections. The higher the concentration of NO_2_, SO_2_, CO, and PM_2.5_, the greater the number of new COVID-19 cases. However, the intensity and dynamic of the causations are different depending on the pollutant being considered. Both NO_2_ (xmap skill = 0.63) and CO (xmap skill = 0.58) have a strong and persisting effect on COVID-19 cases, whereas SO_2_ and PM_2.5_ have a lower (xmap skill = 0.36) and more sporadic impact. The difference in dynamic can be explained by the nature of each pollutant. While most sources of NO_2_ and CO in an urban environment are anthropogenic fossil fuel combustion activities (e.g., transportation emissions, power plants), the SO_2_, apart from traffic, might also originate from other industries (e.g., metallurgy) and volcano emissions in the Andean region [69]. At the same time, in an urban environment a complex-chemistry PM_2.5_ might come from traffic, industries and even natural sources, or over time from precursor contaminants [70]. This makes the latter pollutant quite different from the first three, although it has shown a close correlation with traffic emissions in this fast-developing capital city at high elevation [53].

These findings align with previous studies that have identified a significant association between urban pollution and COVID-19 cases [26,44,49,[71], [72], [73], [74], [75]]. Several factors contribute to this relationship. Firstly, exposure to poor air quality can lead to a reduction in immune defense mechanisms [76], making individuals more susceptible to viral infections. Moreover, it has been suggested that the virus can utilize particulate matter air pollutants as a medium for support and transport, allowing it to persist in the atmosphere and spread from one person to another [75,77]. Understanding the interplay between air pollution and the immune system provides valuable insight into the potential mechanisms behind the higher incidence and severity of COVID-19 cases in polluted areas. By compromising the immune system and serving as a vector for viral dissemination, ambient pollutants may contribute to the increased vulnerability of individuals and the broader transmission of different viruses [76].

In contrast, the effect of O_3_ on the virus differs from the other criteria pollutants. It does not affect COVID-19 infection, but it does affect the mortality rate (xmap skill = 0.36). Unlike the other atmospheric contaminants, this secondary photochemical pollutant seems to have an inhibiting effect on COVID-19-related deaths. This result is supported by the studies showing a chemical degradation of the pathogen agents by an ozone treatment [[78], [79], [80]]. A new contribution of our work is showing that areas of elevated concentrations of O_3_ could be experiencing reduced consequences of the virus on the lethality rate.

The outcome of the CCM analysis reveals a weaker, more contrasted effect of meteorology than pollution on COVID-19. Apart from temperature, the other two meteorological parameters (wind speed and relative humidity) do not seem to have a significant effect on COVID-19 infection. Even if temperature is identified as a causer of contagion, its impact is relatively low (xmap skill = 0.23) compared to air quality. The average interaction strength shows that increasing temperatures result in a reduction in the number of cases. Nevertheless, this general interpretation has to be modulated based on the dynamic of the interaction, which frequently reverses its sign from positive to negative values. This result is supported by other studies that show that a high daily temperature tends to reduce the amount of COVID-19, even if this effect is dependent on the diurnal temperature range [[81], [82], [83]].

On the other hand, our results indicate that meteorology mostly affected the excess deaths. Warm, windy and dry meteorological conditions tend to reduce the mortality rate whereas cold, windless and wet weather is associated with an increase in COVID-19-related deaths. Even if the mechanisms underlying the effect of meteorological parameters on COVID-19 remain unclear, several hypotheses can be arrived at. The negative effect of temperature on the disease can be explained by a thermic degradation of the virus [84]. Cold weather may promote an immunodeficiency, which increases the opportunity for the virus to infect and multiplicate in the host organism [34] or simply cause respiratory and/or cardiovascular distress contributing to deterioration in COVID-19 patients [49,85,86]. The ventilation effect of WS can contribute to cleaning and purifying the planetary boundary layer and is consequently associated with a lower risk of COVID-19 [87]. Conversely, an elevated RH is characterized by water droplets suspended in the air, which prolongs survival time and sustains the transportation of the virus [88]. The observation of the interaction strength for this last parameter reveals a complex relationship with the mortality rate. The instantaneous analysis of the interaction indicates a change in the sign of the causality. This non-linear behavior may explain some contradictory results reported in the literature regarding positive [89], negative [90], or even no correlations [91] between RH on COVID-19. This intrinsic dynamic characteristic of a natural ecosystem justifies the use of CCM to capture the evolution of the causation, which can be masked or reverse its direction depending on the period of time being considered.

Several interpretations can be proposed to explain the temporal variation in the causal effect of the studied variables on COVID-19. Regarding the impact of the pollutants on new cases, the modulation of the interaction strengths can be explained by the fact that the concentration of the air contaminants changes frequently over time. Because the terrain of Quito is complex (Andean mountains), its air quality is unstable with a succession of peaks and drops when it comes to atmospheric pollution [92]. This variation seems to have directly impacted the spread of COVID-19 in the city, considering that the time-delayed causal interaction between most of the pollutants and the new cases is less than one week, according to our model. Similarly, the instability of the weather conditions can explain the variation of the *S*-map coefficient for the meteorological factors. The high elevation of the city strongly impacts the meteorological conditions, with frequent episodes of heavy rain and sun in one day, especially during the wet season [53]. Considering that previous studies have reported a non-linear impact of temperature and relative humidity on COVID-19 [82,83], the large amplitude of variation in the daily value of these variables may be the reason for the complex interaction between meteorology and COVID-19 dynamics in this part of the world.

**Fig. 9:**
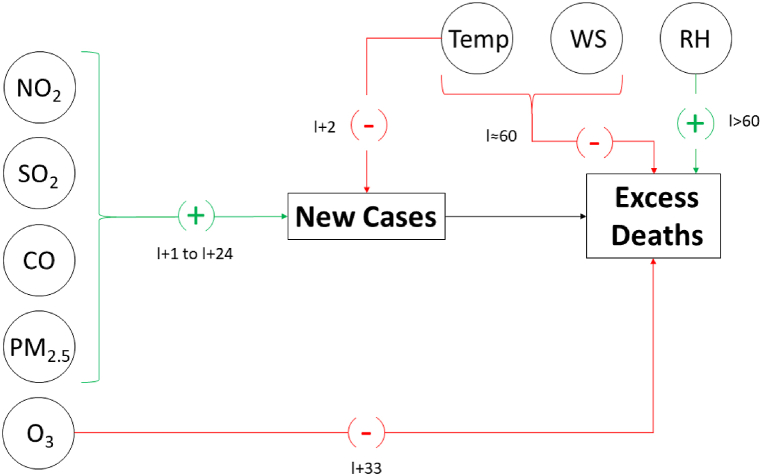
Causal network model. The sign and latency (l) of the effects of the environmental variables on the COVID-19 pandemic are indicated by green and red arrows to represent boosting and inhibiting interactions, respectively. (For interpretation of the references to colour in this figure legend, the reader is referred to the Web version of this article.)

Fig. 9 depicts the resulting causal network based on the outcome of the CCM analysis. It is important to mention that the purpose of this diagram is to provide a general model by which to synthetize the main findings of our study. In that sense, it represents a simplification of the global interactions occurring during the pandemic in the high-elevation tropical capital city of Ecuador. However, as previously described, the details of the interaction dynamic are more complex when they are analyzed at a higher resolution of time scale. Even so, the model shows that four out of five air pollutants are identified as boosters of the COVID-19-related infections. Relative humidity is the only meteorological parameter that contributed to an increase in the lethality of the virus among the inhabitants of Quito. On the other hand, high temperature and wind speed tended to reduce the impact of SARS-CoV-2 on the population for both COVID-19-related deaths (Temp and WS) and COVID-19-related cases (Temp only). Finally, ozone is the only studied criteria pollutant that seems to have a protective effect on the fatal consequences of the coronavirus.

In that sense, our results align with and complement existing knowledge about the influence of air pollution and meteorology on COVID-19. As the pandemic evolved, research studies showed evidence of a correlation between poor air quality and an increase in COVID-19 cases and mortality [[93], [94], [95]]. In terms of NO_2_, we see a very strong positive causation on the number of new COVID-19 cases. Even if a correlation between NO_2_ and COVID-19 spread has already been observed in previous studies [33,44,49], our work shows a quicker effect than what has been reported in the literature [96]. While our analysis captures a causality of SO_2_ on COVID-19 infections, no studies report a direct increase in cases due to this pollutant, but they do report an association of the gas with increased mortality by COVID-19 [46]. The causal effect of PM_2.5_ on COVID-19 cases is also supported by the correlation analysis of other studies [32,33,44,45,74,97]. However, our results point again toward a shorter time-delay of this causal relationship than what was previously supposed [96]. On the other hand, the causation of CO aligns with the correlation analyses on COVID-19 transmissibility in terms of both sign and time lag [71]. The most unexpected result relates to ozone. O_3_ is a highly reactive gas that may be harmful to human health [98]. Nevertheless, our results point toward the benefits of its oxidative effect on COVID-19 and, consequently, the properties it has that can reduce the pervasiveness of the virus in the environment. Finally, the impact of the meteorological parameters on COVID-19 was also suggested to be non-linear and may vary by country [39]. It is confirmed by another study that shows a U-shaped relationship between temperature and mortality [49]. In our work, we reveal that the temperature has a negative causal effect on both new COVID-19 cases and the mortality rate. Relative humidity causes an increased risk of mortality due to COVID-19, which is supported by previous research [88]. As to wind speed, we found a long-term causal effect on the excess deaths, which aligns with the idea that improving air circulation reduces the risk of transmission of SARS-CoV-2 [40].

Before we conclude, it is important to stress that the generalization of our causal network model is obviously limited to the case study from which the data were collected (Quito, Ecuador). Also, it is crucial to remember that inferring causality in non-randomized controlled trials, like the ecological conditions presented here, is not free from failures. CCM is sensitive to the quality of the data collection. Noise in the process or measurement can lead to over connections (bidirectional causality) or under connections (false negative) [62]. However, both the process and the measurements in our study have been carefully controlled, which ensures a high level of confidence in the causal inferences. Furthermore, inferring causality in an observational study may raise the question of confounding variables that might influence the identified causal relationships. Apart from the fact that CCM is less sensitive to this issue than a correlation analysis, our results are based on the rigorous verification of all possible criteria (positive xmap, converging xmap, surrogate test, and prediction lag test) to reduce drastically the risk of false causal inferences. In particular, our analysis of the time-delayed causal interactions makes the causation more robust and accounts for confounders, as demonstrated in recent studies [[99], [100], [101]].

Our causal network underscores the intertwined nature of urban pollution, weather patterns, and COVID-19 dynamics. Urban strategies can harness these insights to mitigate viral spread by reducing pollutants like NO_2_, CO, PM_2.5,_ and SO_2_. Seasonally tailored interventions, based on new knowledge of the impact of climatic conditions on the virus, can optimize resource deployment. Public health in the equatorial regions is largely impacted by the existence of wet and dry seasons. During the wet season, we generally observe cooler temperatures paired with higher relative humidity. Conversely, the dry season is characterized by warmer temperatures and lower relative humidity. This distinction is significant, because our work demonstrates that both temperature and humidity influence the spread and viability of pathogens like SARS-CoV-2. Considering that these seasonal patterns can help public health professionals to anticipate potential outbreaks and devise timely interventions, public awareness campaigns can promote precautionary behaviors, while urban planning can focus on healthcare infrastructure in environmentally strategic areas. Factoring weather into vaccine distribution ensures broader coverage during vulnerable periods. Schools can fine-tune safety protocols based on environmental risks, and the authorities can instore temporary regulations in the community to lower infection rates. Collectively, these insights can pave the way for enhanced public-health preparedness and nuanced management of COVID-19 in urban settings.

Overall, our results align with most of the previous findings on the topic and the causations detected by CCM can be explained by epidemiological mechanisms. The dynamic modeling chosen to identify the causality accounts for a modulation of the interactions over time, with a positive, neutral, or negative effect depending on the temporal window being considered, which is the most appropriate approach by which to analyze ecosystems. Considering the properties of the causation method used and the coherence of our results with the outcomes of other studies, the present article provides a relevant model for a comprehensive assessment of the external drivers involved in the epidemiological consequences of SARS-CoV-2. Future research directions could delve into the intertwined relationship between human mobility and its subsequent impacts on pollution and COVID-19 cases. Enhancing the robustness of the findings might involve comparing the results from the CCM method with other causal analytics methods like Granger causality. Beyond COVID-19, the CCM technique could be applied in relation to other health challenges such as malaria or dengue to reveal, perhaps, causal patterns tied to meteorological or climatic events. Additionally, examining the nuances of disease spread by factoring in specific age and socio-economic conditions can offer deeper insights. Complementing the model with variables like urban infrastructure and public health measures, and assessing regional and seasonal variations of these relationships could also provide a more holistic understanding of the multifaceted interactions influencing disease dynamics.

## Data availability

The original contributions presented in the study are publicly available. The source data can be found here: https://doi.org/10.13140/RG.2.2.10907.85286.

## Ethics declaration

Review and/or approval by an ethics committee was not needed for this study because it involves information that is freely available in the public domain.

## CRediT authorship contribution statement

**Yves Rybarczyk:** Writing – review & editing, Writing – original draft, Visualization, Validation, Methodology, Investigation, Formal analysis, Data curation, Conceptualization. **Rasa Zalakeviciute:** Writing – review & editing, Writing – original draft, Validation, Investigation, Data curation. **Esteban Ortiz-Prado:** Writing – review & editing, Writing – original draft, Investigation.

## Declaration of competing interest

The authors declare that they have no known competing financial interests or personal relationships that could have appeared to influence the work reported in this paper.

## Figures and Tables

**Fig. A 1:**
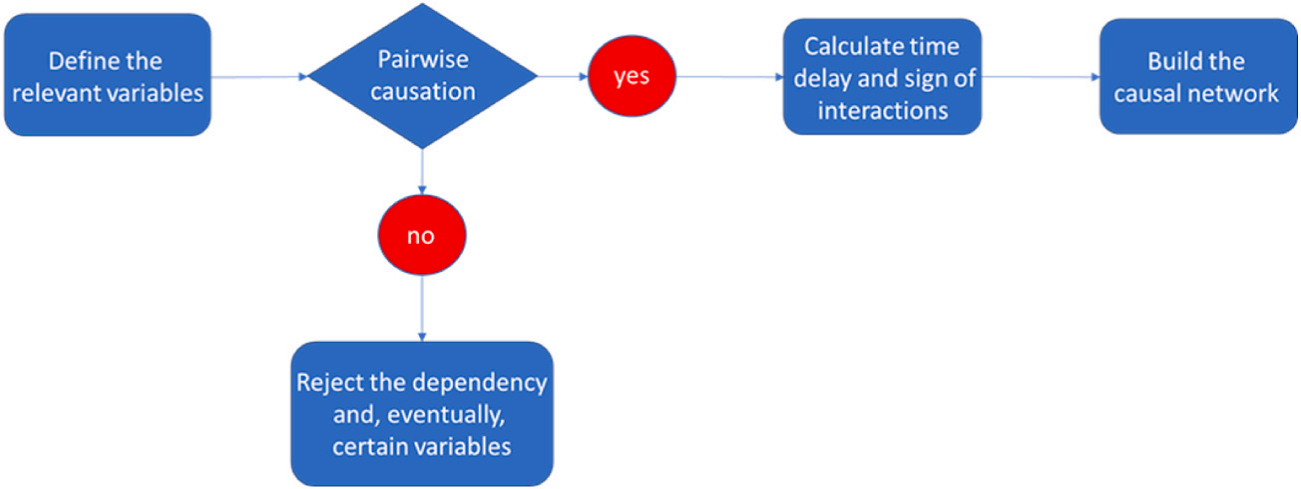
Flowchart of the study.

**Fig. B.1:**
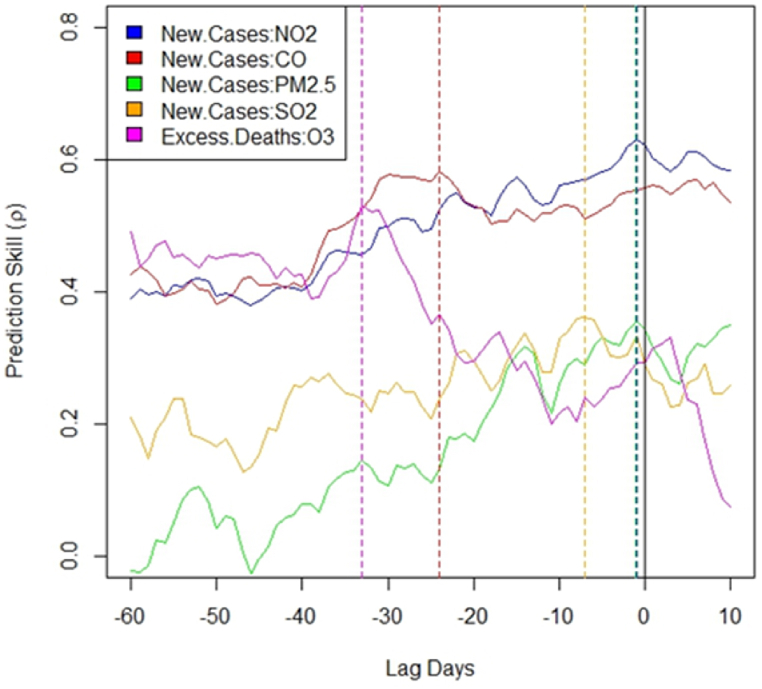
Causal lags and optimal cross mapping between pollution and epidemiological variables (COVID-19 cases and excess deaths). Past and future are identified by negative (−1 to −60) and positive (1–10) values of lag days, respectively. The broken lines indicate the lag days corresponding to the highest prediction skill (ρ) for each air contaminant. It is to note that the xmap skill peaks for NO2 (blue broken line) and PM2.5 (green broken line) overlap at −1 day of lag time

**Fig. B 2:**
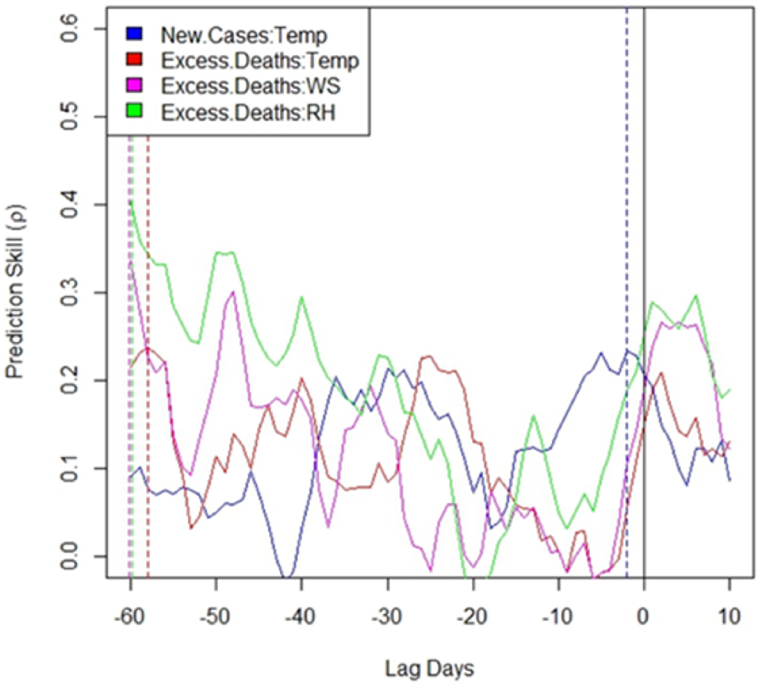
Causal lags and optimal cross mapping between meteorological and epidemiological variables (COVID-19 cases and excess deaths). The broken lines indicate the lag days corresponding to the highest prediction skill (ρ) of each meteorological factor. It is to note that the xmap skill peaks for Excess.Deaths:WS (magenta broken line) and Excess.Deaths:RH (green broken line) are both represented at −60 lag days, because of the maximum range of time delay considered in this study
